# Oestrogen receptor *β* and neoadjuvant therapy with tamoxifen: prediction of response and effects of treatment

**DOI:** 10.1038/sj.bjc.6603082

**Published:** 2006-04-18

**Authors:** W R Miller, T J Anderson, J M Dixon, P T K Saunders

**Affiliations:** 1 Breast Unit Research Group, University of Edinburgh, Western General Hospital, Edinburgh EH4 2XU, UK; 2 MRC Human Reproductive Sciences Unit, Centre for Reproductive Biology, 49 Little France Crescent, Edinburgh EH16 4SB, UK

**Keywords:** breast, oestrogen receptor, ER*β* varient

## Abstract

In order to elucidate the relative importance of oestrogen receptor (ER)*α*, ER*β* and an ER*β* variant (ER*β*2/*β*cx) in the response of breast cancers to tamoxifen, tumour levels of each receptor were assessed in 36 patients before and after 3 months of neoadjuvant treatment with tamoxifen (20 mg daily). All patients were postmenopausal women presenting with large ER*α*-positive breast cancers. Clinical response to treatment was assessed by tumour volume changes as determined from sequential ultrasounds and pathological response by comparison of the tumour morphology before and after treatment. Of 33 cases, 23 (70%) were classified as having a clinical response and 16 (48%) as having a response pathologically. All tumours stained positively for ER*α* and ER*β* and 15 out of 33 (45%) for ER*β*2/*β*cx. There were no significant differences in quantitative expression of any receptor between tumours that subsequently responded and that did not, whether response was assessed clinically or pathologically. Tamoxifen treatment was associated with a decrease in ER*α*, but an increase was the most frequent change (17 out of 33) in ER*β*, and no consistent change was evident in staining of the ER*β*2/*β*cx variant. In summary, ER*β*1 and ER*β*2/*β*cx variant protein are detected in ER*α*-positive breast tumours but their expression is not associated with a response to tamoxifen. Differential changes in ER*α* and ER*β* were seen with treatment.

The anti-oestrogen tamoxifen has a central place in the treatment of breast cancer. However, many tumours appear refractory to the drug and there is a need to discover predictive markers that can accurately identify hormone responsive tumours. In this setting, oestrogen receptor (ER)*α* is the single most informative marker, receptor-negative tumours rarely benefiting from endocrine therapy (Miller, 1996). However, although responses are largely restricted to ER*α*-positive tumours, only between 60 and 70% of these cancers shrink with treatment. Hence, there is a requirement for additional markers to improve discrimination. Interest in the role played by receptors for oestrogen in breast cancer was revitalised by the discovery of a second form of oestrogen receptor, now named ER*β* (Kuiper *et al*, 1996; Mosselman *et al*, 1996). In particular, it is notable that cell-based studies have suggested that coexpression of ER*β* in ER*α*-positive cells may modulate the ability of the cells to respond to oestrogens (Hall and McDonnell, 1999; Strom *et al*, 2004) and studies using mice with targeted disruption of the ER*β* gene have endorsed this idea (Weihua *et al*, 2000; Lindberg *et al*, 2003). In the light of these observations, the suggestion has been made that the level of expression of ER*β* in ER*α*-positive breast cancers might modify tumour response to anti-oestrogenic action. This could account for resistance to endocrine therapy but it remains a topic of debate (reviewed by Warner *et al*, 2000; Speirs *et al*, 2004). The situation is complicated further by the identification of a number of splice variant isoforms of the human ER*β* gene (Moore *et al*, 1998), the mRNAs for which have been detected in breast cancer tissues and breast cancer cell lines (Fuqua *et al*, 1999; Speirs *et al*, 2000; Poola *et al*, 2002a, 2002b). Studies *in vitro* have suggested that ER*β* isoforms with deletions of selected exons, or with alternative splicing at the C-terminus, may act as dominant-negative inhibitors of full-length ER*α* and/or ER*β* (Ogawa *et al*, 1998b; Inoue *et al*, 2000; Peng *et al*, 2003).

An antibody specific for full-length ER*β*, hereafter referred to as ER*β*1, has been used previously to delineate the pattern of expression of this isoform of the receptor in breast cancer biopsies (Saunders *et al*, 2002b; Carder *et al*, 2005). The present paper has taken advantage of the development of a monoclonal specific for the ER*β*cx/*β*2 splice variant (Saunders *et al*, 2002a) to determine whether expression of this isoform influences response to anti-oestrogen therapy as has been claimed by others (Saji *et al*, 2002a, 2002b).

## MATERIALS AND METHODS

### Patients

All patients were referred between 1992 and 1995 to the Edinburgh Breast Unit and had histologically confirmed diagnosis of breast cancer (Miller *et al*, 1999). The women were postmenopausal, aged between 56 and 80 years, had a large (>3 cm) primary tumour with an ER histoscore >80 (equivalent to ⩾5 Allred score – although a single case was subsequently graded as score 4 by the research lab) or a biochemical score of >20 fmol mg protein^−1^ (on the initial biopsy taken for diagnosis) and had no evidence of distant metastatic disease. None had received prior treatment with hormonal agents for breast cancer or were taking hormone preparations at the time of study. Tumour size was monitored clinically (by calipers) and by breast ultrasound before and at monthly intervals during treatment. Therapy comprised daily administration of tamoxifen (25 mg) for 3 months; four patients electively continued on therapy for a further 3 months. A total of 72 consecutive patients were entered into the study; however, data are presented in 33 for ER*α* and ER*β*1 and the ER*β*2/*β*cx variant because in the remainder either pre- and/or post-treatment tumour blocks were exhausted by use for other studies. The demographics of the investigated cases were not different from the recruited population. The studies were performed with the patients’ informed consent and ethical permission (LREC nos. 2001/8/80 and 2001/8/81).

### Clinical response

Clinical response was usually based on change in tumour volume between pretreatment and 3-month values; however, assessment was made at 6 months in those patients electing for extended treatment. Ultrasound measurement of three orthogonal tumour diameters produced an estimate of tumour volume. Reduction in tumour volumes >25% was regarded as evidence of tumour response; those >50% were categorised as major response (Forouhi *et al*, 1994).

### Pathological response

Histological sections from the initial biopsy and the final surgical excision were assessed for decrease in cancer cellularity and increase in fibrosis gland formation. Where this occurred, the tumour was classified as having a pathological response, and where clear changes in cellularity and/or fibrosis were not apparent, the tumour was graded as no pathological response.

### Tumour

Samples of each breast cancer was obtained by biopsy (before treatment) and by definite surgery (wide local excision or mastectomy) after treatment. Tumours were fixed in 10% neutral buffered formaldehyde for 16–24 h, then stored in 70% (w v^–1^) ethanol before processing into paraffin wax at the Department of Pathology using standard procedures.

### Antibodies

The anti-hER*α* mouse monoclonal antibody (code 1D5) was obtained from Dako (Cambridge, UK). Monoclonal antibodies specific for C-terminal peptides within wild-type human ER*β* (hER*β*1, wild type; accession AB006590; Ogawa *et al*, 1998a; Serotec UK MCA1974S) as well as one of the variant isoforms of hER*β* known as hER*β*2/*β*cx (accession AB006589; Moore *et al*, 1998; Ogawa *et al*, 1998b; Serotec UK, MCA 2279S) were prepared using standard methods as described previously (Saunders *et al*, 2000, 2002a). Specificity for the ER*β* isotype to which they were directed has been confirmed on Western blots using recombinant proteins (see Figure 2 in Saunders *et al*, 2002a). Neither antibody showed any crossreactivity against ER*α* (Saunders *et al*, 2002a). These antibodies have been used previously to determine the patterns of expression of ER*β*1 in cancers of the breast (Saunders *et al*, 2002b; Carder *et al*, 2005) and prostate (Torlakovic *et al*, 2002) as well as in a variety of non-malignant adult tissues (Saunders *et al*, 2000, 2002a; Critchley *et al*, 2002; Gaskell *et al*, 2003).

### Immunohistochemistry

Sections (4 *μ*m) were mounted on Superfrost coated slides (BDH, Poole, Dorset, UK), dewaxed and rehydrated in gradient alcohols and distilled water before staining with specific antibodies as outlined below.

#### Anti-ER*α*

All staining for ER*α* was carried out in the Pathology Department of the Western General Hospital. An endogenous biotin block was carried out by applying 100 *μ*l egg white blocking solution for 30 min. Anti-ER*α*Dako was diluted 1 in 50 in biotin diluent for primary antibodies (PBS, goat serum and d-biotin), and applied to the sections for 60 min at room temperature. The secondary antibody, biotinylated anti-mouse Ig (Vector Laboratories, Peterborough, Cambridgeshire, UK) was diluted 1 : 2000 in ‘background reducing diluent’ (Dako) and applied to the sections for 30 min at room temperature. The tertiary system (ABC-HRP, Dako) was applied as per the manufacturer's instructions for 30 min at room temperature. The tissue was visualised by immersing sections in 3,3′-diaminobenzidine tetrahydrochloride (DAB) for 5 min. Sections were counterstained using Mayers haematoxylin (Sigma-Aldrich, Poole, Dorset, UK), dehydrated through gradient alcohols and mounted.

#### Anti-ER*β*

Tissue sections were dewaxed in Histoclear (National Diagnostics, Atlanta, GA, USA) and rehydrated in descending grades of alcohol to dH_2_O. Antigen retrieval was carried out by pressure cooking in 0.05 M glycine 0.01% EDTA pH 3.5 for 3 min setting 2 (Tefal, Nottingham, UK) and sections left to stand undisturbed for 20 min. Sections were blocked for 30 min in normal rabbit serum (NRS; Diagnostics Scotland, Edinburgh, Scotland, UK) diluted 1 : 4 in TBS containing 5% BSA (NRS/TBS/BSA), rinsed briefly in TBS and an avidin–biotin block performed using reagents from Vector (Petersborough, UK). Anti-ER*β* antibodies were diluted in NRS/TBS (ER*β*1, 1 in 20; ER*β*cx/*β*2 1 in 40) and incubated on sections overnight at 4°C. Sections were washed twice for 5 min each in TBS and incubated with biotinylated rabbit anti-mouse immunoglobulin (Dako) diluted 1 : 500 in NRS/TBS/BSA. Bound antibodies were visualised by incubation with DAB (liquid DAB cat K3468, Dako); the DAB was added to sections at 8 s intervals and the colour allowed to develop for exactly 3 min (ER*β*1) or 5 min (ER*β*2/*β*cx). Control sections previously used to determine the dilutions of antibodies were included in all experimental runs. Sections were counterstained with haematoxylin.

Images were captured using an Olympus Provis microscope (Olympus Optical Co, London, UK) equipped with a Kodak DCS330 camera (Eastman Kodak Co, Rochester, NY, USA).

### Quantitation of immunohistochemical staining

Quantitation was based on a scoring system reported in detail previously (Allred *et al*, 1998; Leake *et al*, 2000). This method is based on a composite additive score of intensity 0–3 and the proportion of malignant epithelial cells staining 0–5. This gives a range of 0–8 for each tissue. Statistical analysis was carried out using the Wilcoxon matched-pairs signed-ranks test, as this is more sensitive than the Student's *t*-test for small numbers of samples.

## RESULTS

### Response

Of the 33 patients studied, 23 (70%) were classified as having a clinical response and 16 (48%) as having a response in tumour pathology. Although the majority of clinical responders/non-responders had corresponding changes in tumour pathology, one tumour that did not change in tumour volume with treatment showed significant changes in tumour morphology; conversely, eight tumours shrinking clinically with treatment did not change their morphological appearance.

### Oestrogen receptor *α*

All patients were required to have ER*α*-positive tumour in order to be eligible for the study. There was no quantitative difference in the initial staining score of tumours that either responded or did not, clinically or morphologically (data not shown). Median (range) was 7 (6–8) for responding tumours and 7 (4–8) for non-responders (Table 1A).

Comparison of biopsies taken before and during treatment with tamoxifen showed that ER*α* category scores decreased in all but six tumours (five tumours were unchanged and one increased). Although only one score fell to 0, the difference between pre- and treated tumour was highly significant (*P*<0.0001 by paired Wilcoxon test). No significant differences in change of score were apparent between tumours that responded and that did not, whether response was assessed clinically or by morphology (Table 1).

### Oestrogen receptor *β*1

All tumours stained positively with scores ranging from 5 to 8. Although the median value was similar in cancers responding and not responding clinically (median 7, range 5–8 in responding tumours, and median 7, range 6–8 in non-responding cancers) because of the difference in the distribution of values, levels were significantly higher in non-responding tumours (*P*=0.03 by paired Wilcoxon test). The levels of ER*β*1 were not significantly different in tumours that morphologically changed with treatment as compared with those that did not. There was no correlation between ER*β*1 and ER*α* scores and expressing results as the ratio of ER*α* to ER*β*1 failed to increase discrimination between responding and non-responding tumours (Table 2).

In contrast to ER*α*, tamoxifen treatment tended to be associated with an increase in the staining intensity of ER*β*1, with 17 of 33 having a higher staining score after treatment. This difference in the staining pattern of ER*β*1 with treatment is significantly different from that observed in ER*α* (*P*=0.001). However, the pattern of change in the ER*β*1 score with treatment did not differ according to response, whether this was assessed clinically or by tumour pathology.

### Oestrogen receptor *β*2/*β*cx splice variant

**Figure 1:**
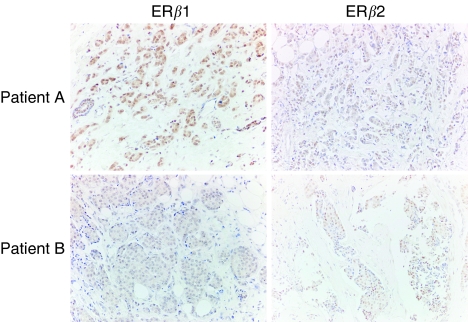
In breast cancer biopsies, ER*β*2/*β*cx immunostatus did not parallel that of ER*β*1. Results from two patients before therapy are shown. Note that in patient A, the level of expression of ER*β*1≫ER*β*2/*β*cx, whereas in patient B, ER*β*1&lt⩽ER*β*2/*β*cx. Magnifications × 40.

Expression of the ER*β*2/*β*cx variant was detected by immunohistochemistry in 15 of 33 tumours (45%). Before therapy, the level of expression of ER*β*2/*β*cx did not relate to expression of ER*α* or ER*β*1, and examples of divergent results are shown in Figure 1. Note that as ER*β*1 immunoexpression was found in all tumours, 55% were ER*β*1 positive/ER*β*2/*β*cx negative. There was no difference in the status/level of expression of ER*β*2/*β*cx variant between tumours responding to and those that did not to tamoxifen (Table 3). Expressing results as a ratio with ER*α* or ER*β*1 failed to increase discrimination between responding and non-responding tumours (results not shown).

**Figure 2:**
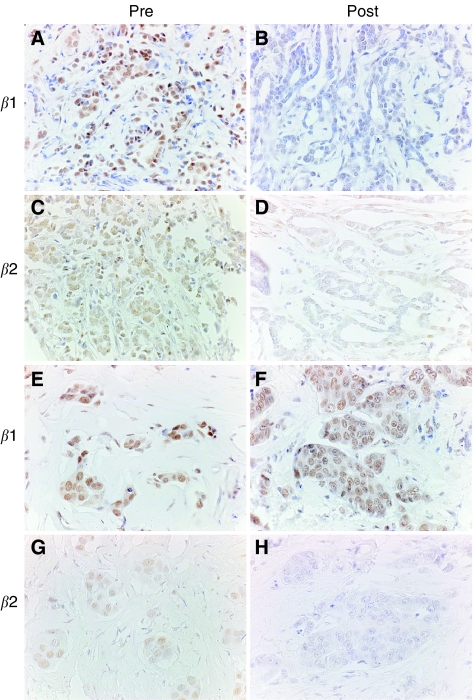
Immunohistochemical localisation of ER*β* proteins to breast biopsies obtained before and after treatment with tamoxifen. Results for ER*β*1 (**A**, **B**, **E**, **F**) and the ER*β*2/*β*cx variant (**C**, **D**, **G**, **H**) are shown for two patients only one of whom (**A**–**D**) showed a positive clinical response to therapy. In both patients, ER*β*1-positive malignant cells were present before treatment (**A**, **E**), but whereas immunoexpression was reduced in the patient who responded to therapy (compare **A** and **B**) there was no reduction in expression in the other patient who did not exhibit a clinical response (compare **D** with **F**). In both patients, expression of ER*β*2/*β*cx was reduced (compare **C** and **D**; **G** and **H**). Magnifications, × 40.

Treatment with tamoxifen resulted in decreased expression of ER*β*2/*β*cx in 11 cases, increased expression in six tumours and no changes in 16. Changes in expression were not related to response or to changes in ER*α* and ER*β*1 (Figure 2).

## DISCUSSION

We have used well-characterised monoclonal antibodies (Critchley *et al*, 2002; Saunders *et al*, 2002a) to compare the pattern of expression of two isoforms of ER*β*, namely the full-length functional receptor (ER*β*1) and the ER*β*2/*β*cx variant (Moore *et al*, 1998; Ogawa *et al*, 1998b). Oestrogen receptor *β*1 contains a functional steroid binding pocket (Pike *et al*, 1999), an intact AF-2 domain capable of recruiting coactivators (Klinge, 2000) and is capable of inducing gene transcription *in vitro* (Paech *et al*, 1997; Barkhem *et al*, 1998; Sierens *et al*, 2004). In contrast, ER*β*2/*β*cx lacks 61 amino acids normally found at the C-terminus of ER*β*1 and instead contains novel 27 amino acids that do not encode a functional AF-2 domain (Ogawa *et al*, 1998b). Studies *in vitro* have shown that ER*β*2/*β*cx-containing constructs do not bind oestradiol and that neither can they induce gene expression via oestrogen response elements (EREs) in reporter assays (Ogawa *et al*, 1998b; Peng *et al*, 2003; Sierens *et al*, 2004). Interest in determining whether ER*β*2/*β*cx is expressed in breast and other cancers has been fuelled by studies using transfected cells that have claimed that coexpression of ER*β*2/*β*cx with ER*α* results in reduced activation of ERE-containing reporter constructs (Ogawa *et al*, 1998b; Peng *et al*, 2003).

In the current study, we did not find any correlation between the intensity of immunoexpression of ER*β*1 and ER*β*2/*β*cx in breast cancer biopsies taken before treatment, with a higher proportion of the tissues being immunopositive for ER*β*1 than for ER*β*2/*β*cx. This finding was unexpected as both the proteins are encoded by the same gene and are identical in sequence apart from alternative splicing of alternative eighth exons. We have previously noted differences in the pattern of expression of ER*β*1 and ER*β*2/*β*cx in non-malignant tissues including the testis and endometrium (Critchley *et al*, 2002; Saunders *et al*, 2002a) and therefore do not believe that this finding is associated with the development of malignancy although it does raise questions as to the mechanisms controlling splicing of the human ER*β* gene. We detected ER*β*2/*β*cx protein in 45% of the tumours. In other studies in which expression of ER*β*2/*β*cx has been assessed using different ER*β*2/*β*cx-specific antibodies, the protein has been detected in 48% (Saji *et al*, 2002b; Palmieri *et al*, 2004) or 56% (Omoto *et al*, 2002) of the tissues examined, which is in general agreement with our own findings. Comparison with other studies that have reported the incidence of ER*β* immunostaining is not possible because they used antibodies that would not discriminate between ER*β*1 and ER*β*2/*β*cx variants (Jarvinen *et al*, 2000; Skliris *et al*, 2001).

Functional studies have previously claimed that the agonist activity of tamoxifen was ER*α* dependent (Watanabe *et al*, 1997). However, a recent study in which the conformations adopted by ER*α* and ER*β* following binding to tamoxifen were investigated has resulted in a revised model for binding that includes interactions with both ER*α* and ER*β* (Heldring *et al*, 2004). It is notable that in the current study, tamoxifen treatment had an apparent impact on the levels of expression of both ER*α* and ER*β*, but that the effects were opposite to each other, with levels of ER*α* declining and those of ER*β*1 increasing.

These cell-based studies provide a rationale for considering whether the expression of ER*β*1 and/or ER*β*2/*β*cx in malignant cells within the breast can influence the response of the tissue to therapy with tamoxifen. Although there is a general consensus that the presence of ER*α* predicts response to tamoxifen (ER*α*-negative tumours rarely respond), the literature relating to ER*β* and response to tamoxifen is confusing and conflicting. In part, this is because studies have been performed in two different settings, giving tamoxifen either as an adjuvant to surgery and measuring recurrence rates/times (Murphy *et al*, 2002; Davies *et al*, 2004; Esslimani-Sahla *et al*, 2004; Hopp *et al*, 2004; O’Neill *et al*, 2004) or as neoadjuvant treatment and monitoring changes in the size of the primary tumours. In general, the latter studies are more applicable to tumour sensitivity to therapy, as recurrence in the adjuvant setting is determined not only by response to systemic treatment but also by the extent of micrometastatic disease and inherent aggressiveness of the tumour. In this respect, the most substantive neoadjuvant studies to date examining effects of the closely related anti-oestrogen, toremifene, in 38 cases of preoperatively and 20 cases of postoperatively indicated that, as in the present study, response was independent of ER*β* levels or changes (Cappelletti *et al*, 2004). Two other studies have investigated ER*β*2/*β*cx. One reported 23 patients who were treated neoadjuvantly and found a statistically significant association between the presence of ER*β*2/*β*cx and response to tamoxifen (*P*=0.04) but the group was unusual in that all three ER*α*-negative tumours also responded to treatment. The other study (Saji *et al*, 2002b) evaluated 18 cases and found ER*β*2/*β*cx expression to be associated with less chance of response to tamoxifen. Given that the present study failed to find either a positive or a negative association between ER*β*2/*β*cx and response to tamoxifen, it seems unlikely that ER*β*2/*β*cx will be predictive of response in individual cases.

In the current study, although ER*β*1 protein was detected in all tumours, there was no correlation between the levels in those that responded to treatment and those that did not. Furthermore, there was no difference in the status/level of expression of ER*β*2/*β*cx between tumours responding to and those that did not to tamoxifen. In conclusion, to date, there is as yet no consensus among the different studies in which response to tamoxifen has been correlated with expression of ER*β* and/or the ER*β*2/*β*cx variant as to whether measuring ER*β* expression in ER*α*-positive breast cancers is likely to be informative as regards response to anti-oestrogen therapy.

## Figures and Tables

**Table 1:** Oestrogen receptor *α*

**(A) Immunohistochemical score in tumours responding (Resp) and not responding (Non-R) to tamoxifen^a^**				
**Score**	**4**	**6**	**7**	**8**
Total	1	2	17	13
Resp (Clin)		1	13	9
Non-R (Clin)	1	1	4	4
Resp (Path)		1	10	5
Non-R (Path)	1	1	7	8
				

**(B) Change in immunohistochemical score with tamoxifen treatment^a^**				
	**Decrease**	**No change**	**Increase**	
Total	27	5	1	
Clin Resp	18	4	1	
Clin Non-R	9	1	0	
Path Resp	12	3	1	
Path Non-R	15	2	0	

a No significant differences between responding and non-responding tumours.

**Table 2:** Oestrogen receptor *β*1 (wild type)

**(A) Immunohistochemical score in tumours responding (Resp) and not responding (Non-R) to tamoxifen^a^**				
**Score**	**5**	**6**	**7**	**8**
Total	1	8	19	5
Resp (Clin)	1	7	14	1
Non-R (Clin)	0	1	5	4
Resp (Path)	0	6	8	2
Non-R (Path)	1	2	11	3
				

**(B) Change in immunohistochemical score with tamoxifen treatment^b^**				
	**Decrease**	**No change**	**Increase**	
Total	8	8	17	
Resp (Clin)	5	4	14	
Non-R (Clin)	3	4	3	
Resp (Path)	4	5	7	
Non-R (Path)	4	3	10	

a Significant difference between tumours responding and not responding clinically, *P*<0.015 by *χ*^2^ test for trends.

b No significant differences between responding and not responding tumours.

**Table 3:** Oestrogen receptor *β*2/*β*cx (variant)

**(A) Immunohistochemical score in tumours responding (Resp) and not responding (Non-R) to tamoxifen^a^**				
**Score**	**0**	**4**	**5**	**6**
Total	18	4	9	2
Resp (Clin)	12	3	7	1
Non-R (Clin)	6	1	2	1
Resp (Path)	7	1	7	1
Non-R (Path)	11	3	2	1
				

**(B) Change in immunohistochemical score with tamoxifen treatment^a^**				
	**Decrease**	**No change**	**Increase**	
Total	11	16	6	
Resp (Clin)	8	10	5	
Non-R (Clin)	3	6	1	
Resp (Path)	5	8	3	
Non-R (Path)	6	8	3	

a No significant differences between responding and non-responding tumours.
